# Sequencing of 15 622 gene‐bearing BACs clarifies the gene‐dense regions of the barley genome

**DOI:** 10.1111/tpj.12959

**Published:** 2015-09-21

**Authors:** María Muñoz‐Amatriaín, Stefano Lonardi, MingCheng Luo, Kavitha Madishetty, Jan T. Svensson, Matthew J. Moscou, Steve Wanamaker, Tao Jiang, Andris Kleinhofs, Gary J. Muehlbauer, Roger P. Wise, Nils Stein, Yaqin Ma, Edmundo Rodriguez, Dave Kudrna, Prasanna R. Bhat, Shiaoman Chao, Pascal Condamine, Shane Heinen, Josh Resnik, Rod Wing, Heather N. Witt, Matthew Alpert, Marco Beccuti, Serdar Bozdag, Francesca Cordero, Hamid Mirebrahim, Rachid Ounit, Yonghui Wu, Frank You, Jie Zheng, Hana Simková, Jaroslav Dolezel, Jane Grimwood, Jeremy Schmutz, Denisa Duma, Lothar Altschmied, Tom Blake, Phil Bregitzer, Laurel Cooper, Muharrem Dilbirligi, Anders Falk, Leila Feiz, Andreas Graner, Perry Gustafson, Patrick M. Hayes, Peggy Lemaux, Jafar Mammadov, Timothy J. Close

**Affiliations:** ^1^ Department of Botany and Plant SciencesUniversity of CaliforniaRiversideCA92521USA; ^2^ Department of Computer ScienceUniversity of CaliforniaRiversideCA92521USA; ^3^ Department of Plant SciencesUniversity of CaliforniaDavisCA95616USA; ^4^ Nordic Genetic Resource CenterSE‐23053AlnarpSweden; ^5^ The Sainsbury LaboratoryNorwich Research ParkNorwichNR4 7UHUK; ^6^ Department of Crop and Soil SciencesWashington State UniversityPullmanWA99164USA; ^7^ Department of Plant BiologyDepartment of Agronomy and Plant GeneticsUniversity of MinnesotaSt. PaulMN55108USA; ^8^ Corn Insects and Crop Genetics ResearchUSDA‐Agricultural Research Service & Department of Plant Pathology and MicrobiologyIowa State UniversityAmesIA50011‐1020USA; ^9^ Leibniz Institute of Plant Genetics and Crop Plant Research (IPK)D‐06466GaterslebenGermany; ^10^ Molefarming Laboratory USADavisCA95616USA; ^11^ Departamento de Ciencias BasicasUniversidad Autonoma Agraria Antonio NarroNarro 1923SaltilloCoah25315México; ^12^ Arizona Genomics InstituteUniversity of ArizonaTucsonAZ85721USA; ^13^ Monsanto Research CenterBangalore560092India; ^14^ USDA‐ARS Biosciences Research LabFargoND58105USA; ^15^ Ronald Reagan UCLA Medical CenterLos AngelesCA90095USA; ^16^ Keck School of MedicineUniversity of Southern CaliforniaLos AngelesCA90033USA; ^17^ Turtle Rock StudiosLake ForestCA92630USA; ^18^ Department of Computer ScienceUniversity of TurinCorso Svizzera 18510149TurinItaly; ^19^ Deptartment of Mathematics, Statistics and Computer ScienceMarquette UniversityMilwaukeeWI53233USA; ^20^ Google Inc.Mountain ViewCA94043USA; ^21^ USDA‐ARSAlbanyCA94710USA; ^22^ Agriculture and Agri‐Food CanadaMordenMBR6M 1Y5Canada; ^23^ School of Computer EngineeringNanyang Technological UniversityNanyang AvenueSingapore639798Singapore; ^24^ Centre of the Region Hana for Biotechnological and Agricultural ResearchInstitute of Experimental BotanySokolovskį 6CZ‐77200OlomoucCzech Republic; ^25^ Hudson Alpha Genome Sequencing CenterDOE Joint Genome InstituteHuntsvilleAL35806USA; ^26^ US Department of Energy Joint Genome InstituteWalnut CreekCA94598USA; ^27^ Baylor College of MedicineJan and Dan Duncan Neurological Research InstituteHoustonTX77030USA; ^28^ Department of Plant Sciences & Plant PathologyMontana State UniversityBozemanMT59717‐3150USA; ^29^ USDA‐ARSAberdeenID83210USA; ^30^ Department of Crop and Soil ScienceOregon State UniversityCorvallisOR97331USA; ^31^ Department of Botany and Plant PathologyOregon State UniversityCorvallisOR97331USA; ^32^ International Cooperation DepartmentThe Scientific and Technological Research Council of TurkeyTunus cad. No: 8006100KavaklidereAnkaraTurkey; ^33^ Swedish University of Agricultural SciencesSE‐750 07UppsalaSweden; ^34^ Boyce Thompson Institute for Plant ResearchCornell University533 Tower RoadIthacaNY14853‐1801USA; ^35^ USDAUniversity of MissouriColumbiaMO65211USA; ^36^ Department of Plant and Microbial BiologyUniversity of CaliforniaBerkeleyCA94720USA; ^37^ Department of Crop & Soil Environmental SciencesVirginia TechBlacksburgVA24061USA; ^38^ Dow AgroSciences LLCIndianapolisIN46268‐1054USA

**Keywords:** Barley, *Hordeum vulgare* L., BAC sequencing, gene distribution, recombination frequency, synteny, centromere BACs, HarvEST:Barley, *Aegilops tauschii*

## Abstract

Barley (*Hordeum vulgare* L.) possesses a large and highly repetitive genome of 5.1 Gb that has hindered the development of a complete sequence. In 2012, the International Barley Sequencing Consortium released a resource integrating whole‐genome shotgun sequences with a physical and genetic framework. However, because only 6278 bacterial artificial chromosome (BACs) in the physical map were sequenced, fine structure was limited. To gain access to the gene‐containing portion of the barley genome at high resolution, we identified and sequenced 15 622 BACs representing the minimal tiling path of 72 052 physical‐mapped gene‐bearing BACs. This generated ~1.7 Gb of genomic sequence containing an estimated 2/3 of all Morex barley genes. Exploration of these sequenced BACs revealed that although distal ends of chromosomes contain most of the gene‐enriched BACs and are characterized by high recombination rates, there are also gene‐dense regions with suppressed recombination. We made use of published map‐anchored sequence data from *Aegilops tauschii* to develop a synteny viewer between barley and the ancestor of the wheat D‐genome. Except for some notable inversions, there is a high level of collinearity between the two species. The software HarvEST:Barley provides facile access to BAC sequences and their annotations, along with the barley–*Ae. tauschii* synteny viewer. These BAC sequences constitute a resource to improve the efficiency of marker development, map‐based cloning, and comparative genomics in barley and related crops. Additional knowledge about regions of the barley genome that are gene‐dense but low recombination is particularly relevant.

## Significance Statement

We provide new genome sequence resources for barley and related species, to improve the efficiency of marker development, map‐based cloning, comparative genomics studies, and to provide insights into genome architecture. Notably, some of the gene‐rich islands in the barley genome deviate markedly from assumption that gene density and high recombination frequency are correlated.

## Introduction

Since Neolithic times, barley has played a major role as a source of food, feed, and beer (Ullrich, 2010). The ability of barley to adapt to marginal environments together with the distinctive grain characteristics make barley a versatile crop that is grown worldwide (reviewed in Muñoz‐Amatriaín *et al*., 2014). However, genes determining these valuable features are contained in a highly repetitive and complex genome almost twice the size of that in humans.

With the advent of next‐generation sequencing (NGS), the barley community envisioned the sequencing of a complete barley genome (Schulte *et al*., 2009). Chromosome sorting by flow cytometry reduced the genome complexity allowing the application of NGS to barley chromosome arms (Doležel *et al*., 2012), which was applied to assemble the detected genes in a synteny‐based virtual linear order (Mayer *et al*., 2011). In 2012, The International Barley Sequencing Consortium (IBSC) released an extensive genome sequence resource that integrated annotated whole‐genome shotgun (WGS) sequences within a physical and genetic framework (IBSC, 2012). Bacterial artificial chromosome (BAC) and BAC‐end sequences assisted the incorporation of WGS sequence data into the physical map, and the integration of the physical and genetic maps. Most of the 6278 sequenced BACs that were included in that work were gene‐bearing BACs identified from the Yu *et al*. (2000) library of cv. Morex. Subsequently, additional anchoring of WGS contigs by POPSEQ (POPulation SEQuencing; Mascher *et al*., 2013) led to an improved coupling of the barley physical map (Ariyadasa *et al*., 2014) to the genetic map. However, resolution was still quite limited as it included only the BAC sequences that were previously published (IBSC, 2012), providing a ‘sequence‐ready’ physical map (Ariyadasa *et al*., 2014).

Sequencing the entire barley genome has been a challenge due to difficulties resolving the abundant and complex repetitive regions during assembly (Stein and Steuernagel, 2014). However, the makeup of the barley genome presents some opportune portals of entry. Estimates indicate that barley gene content is similar to that of the grass model rice, even though the latter is almost 12 times smaller (IRGSP, 2005; IBSC, 2012). Several studies have shown that the >80% repetitive DNA is not randomly distributed across the barley genome and that there are gene‐rich regions with relatively little repetitive DNA which exhibit extensive collinearity with other grasses (e.g. Feuillet and Keller, 1999; Sandhu and Gill, 2002; Varshney *et al*., 2006; Wicker *et al*., 2009). A selective sequencing strategy to target gene‐containing regions of the genome has provided a feasible approach to explore and characterize the genomic features of the gene‐rich portion of barley, the Triticeae model genome.

In an effort that started over a decade ago, the original Yu *et al*. (2000) BAC library constructed from cv. Morex was screened for gene‐containing BACs. Here we present the development of a minimal tiling path (MTP) of 15 711 ‘gene‐bearing’ BACs, summaries of annotated and map‐anchored sequences of 15 622 of these clones, and facile access to this information. An earlier version of the sequence assembly for over 2000 of these MTP BACs was released with the 2012 genome sequence resource publication (IBSC, 2012). Here we provide much‐improved sequence assemblies for those clones, along with all of the remaining MTP of gene‐bearing BAC contigs. These ~1.7 Gb of gene‐rich genomic sequence expand our knowledge of the characteristic features of the gene‐containing regions. Furthermore, this resource will improve the speed and precision of map‐based cloning and marker development in barley and closely related species while supporting ongoing efforts in obtaining a complete reference sequence of barley.

## Results and Discussion

### A physical map of the gene‐containing portion of the barley genome

The barley (cv. Morex) BAC library described by Yu *et al*. (2000) has been extensively used for positional gene cloning (e.g. Wei *et al*., 1999, 2002; Yan *et al*., 2006b; Komatsuda *et al*., 2007), comparative sequence analysis between related species (e.g. Dubcovsky *et al*., 2001; Griffiths *et al*., 2003) and physical mapping (IBSC, 2012; Ariyadasa *et al*., 2014). This library, composed of 313 344 BAC clones representing 6.3× haploid genome coverage, was screened with genic probes to identify a subset of 83 831 ‘gene‐bearing’ BACs (hereafter referred to simply as gene‐bearing BACs or GB‐BACs). Rearrays encompassing these 83 831 BACs were fingerprinted using the high information contig fingerprinting (HICF) method of Luo *et al*. (2003). Among the 72 052 clones that were effectively fingerprinted, 61 454 were assembled into 10 794 contigs (Data S1) using a compartmentalized assembly method (Bozdag *et al*., 2009). This assembly thus had an average of 5.7 BACs per fingerprinted contig (FPC) along with 10 598 singletons. The assembly is available at http://phymap.ucdavis.edu/barley/ in the database ‘Barley Compartmentalized PhyMap V14.’

Minimal tiling path clones were chosen using the FMTP method of Bozdag *et al*. (2013) to reduce redundancy prior to sequencing. The set of MTP clones included a total of 15 711 unique BACs. The MTP presented in this study was computed independently from that generated by the IBSC (2012). Data S1 provides a summary of the origination of the full list of GB‐BACs and these MTP clones.

### Gene‐bearing MTP BAC‐clone sequencing and assembly

To sequence the 15 711 unique BAC clones comprising the gene‐bearing MTP we followed essentially the pooled‐clone combinatorial sequencing method described by Lonardi *et al*. (2013). In brief, eight sets of BAC clones (sets HV3 to HV10) were sequenced using Illumina HiSeq2000. High‐quality reads were assigned (or *deconvoluted*) to individual BAC clones and then assembled BAC‐by‐BAC using Velvet v. 1.2.09 (Zerbino and Birney, 2008). Only *nodes* (i.e., Velvet contigs) with a size of at least 200 bp were used for further analysis in the present work. Assembly statistics for nodes of different minimum sizes are reported in Table S1.

In total, 15 622 BAC assemblies were obtained, which represents 99.4% of all MTP BACs attempted to be sequenced. These BAC assemblies had an average N50 of 23.9 kb and an average L50 of 2.8 nodes (Table 1). Altogether, the assembly generated 1.7 Gb of gene‐rich genomic sequence, which amounts to ~33.3% of the barley genome (ca 5.1 Gb; IBSC, 2012) (Table 1).

**Table 1:** Statistics of the gene‐bearing BAC sequence assembly for nodes ≥200 bp in size

Chr. arm	No. BACs	Avg. no. nodes per BAC	Length of assembled reads (bp)	Avg. BAC length (bp)	Avg. N50	Avg. L50	No. unique HC gene models a	No. unique LC gene models a
1H	1959	19.5	209 800 854	107 096	23 697	2.8	2866	3502
2HS	1048	18.5	111 465 824	106 361	24 703	2.7	1486	1935
2HL	1391	19.1	149 179 441	107 246	22 978	2.7	2241	2529
3HS	862	18.2	92 368 717	107 156	26 301	2.6	1242	1704
3HL	1389	18.8	148 465 120	106 886	24 223	2.7	2132	2576
4HS	862	17.9	94 522 065	109 654	26 577	2.6	1048	1442
4HC	60	15.8	5 866 127	97 769	28 999	2.2	20	40
4HL	1100	18.7	120 519 518	109 563	25 756	2.7	1536	1740
5HS	640	19.1	69 074 495	107 929	24 696	2.8	812	1207
5HL	1623	20.1	173 282 123	106 767	22 285	2.9	2777	3288
6HS	823	19.7	87 025 477	105 742	22 248	2.8	1070	1624
6HL	1113	19.3	120 662 552	108 412	23 922	2.8	1610	1942
7HS	1196	19	129 484 046	108 264	24 580	2.7	1770	2496
7HL	1150	20.1	122 082 348	106 159	23 221	2.8	1734	2182
NA	406	41.6	63 956 654	157 529	16 942	5.2	994	1287
All	15 622	19.7	1 697 755 361	108 677	23 906	2.8	15 707	19 330

N50: length for which the collection of all nodes (contigs) of that length or longer contains at least half of the sum of the lengths of all nodes (contigs) in the BAC assembly.

L50: minimum number of nodes (contigs) accounting for more than 50% of the BAC assembly.

a Gene models hitting 10 or more BACs are not included in the count.

BACs were assigned to barley chromosome arms using a tool called CLARK (CLAssifier based on reduced K‐mers; Ounit *et al*., 2015). Using flow‐sorted materials (Suchánková *et al*., 2006) that were shotgun sequenced and assembled (see Experimental procedures), this approach allocated 15 216 BAC assemblies (97.4% of those sequenced) with high confidence to chromosome 1H or arms of chromosomes 2H–7H (Table 1; Data S1). The number of BACs per chromosome ranged from 1936 for 6H to 2439 for 2H (Table 1). We observed a linear relationship between the number of sequenced BACs per arm and the molecular size of the corresponding barley chromosome arm reported by Suchánková *et al*. (2006) (*r *= 0.953; Figure S1). This outcome indicates that chromosomal gene content in barley is proportional to size. It should be noted that 60 BACs are located in a region that is overlapped by 4HS and 4HL cytogenetic stocks, which we defined as ‘4HC.’ Some of them could be centromere clones (see below). Only 406 BACs could not be assigned to 1H or an arm of any other chromosome. Physical chimerism, or cross‐contamination of cultures or DNA samples during handling seem to be the most likely explanations of assignment failure for these clones, which as a group have anomalous metrics (see Table 1).

Previously predicted barley genes (IBSC, 2012) classified into high confidence (HC) and low‐confidence (LC) were used to annotate BAC assemblies. In total, 17 386 HC and 21 175 LC gene models were found in our BAC sequences. The number of HC genes contained in the 15 622 BACs represents 67% of all annotated HC barley genes (26 159 genes; IBSC, 2012). This value is derived from a large sample size, so it implies that this is the portion of all barley genes represented in these BACs, whether previously annotated or not. We noticed that some gene models generated BLAST alignments to a large number of BACs, so for several calculations we excluded any gene model hitting ten or more BACs (1679 HC and 1845 LC genes; Table S2) since their inclusion would obscure more meaningful information. These gene models most often seem to contain transposable element (TE)‐related sequences. Over 88% of the MTP BACs (13 809 BACs) contained at least one gene model, which indicates that the number of false positives occurring during the identification of gene‐bearing BACs was low.

Our short‐read based sequencing and assembly methods were validated by comparing the assemblies to those from 997 of the same BACs that had been previously sequenced by other institutions using 454/Roche technology (IBSC, 2012). Our assemblies covered, on average, 87.1% of BACs sequences that were generated using 454. Very similar gene densities and gene contents were found in assemblies produced independently by these two approaches (Table 2). Additionally, we compared 14 of our short‐read based BAC assemblies to the complete sequences of the corresponding BACs produced using Sanger sequencing (14 of 50 sequenced by the Joint Genome Institute). On average, the short‐read assemblies covered 88.3% of the complete BAC sequence with a range of 75.2– 98.6%. While this is not 100% coverage, all of the HC gene models (40) and single‐nucleotide polymorphism (SNP) design sequences (31) are included.

**Table 2:** Comparison between gene models found in two different sequence assemblies of 997 BACs. High confidence (HC) and low‐confidence (LC) gene models predicted by IBSC (2012) were considered. A minimum sequence length of 200 bp and an e‐value of 1e^−20^ were the cutoffs used for the BLAST alignments. Numbers do not include gene models hitting ≥10 BACs

Sequencing technology	Avg. HC gene models / BAC	Total unique HC gene models	Avg. LC gene models / BAC	Total unique LC gene models
454^a^	2.96	2604	3.26	2950
Illumina^b^	2.89	2571	3.18	2904
Both	2.83	2489	3.08	2785

a Sequencing institutions for 454 sequencing included IPK Gatersleben, Fritz‐Lipman Institute in Jena and Eurofins Scientific, and were published in IBSC (2012).

b Sequencing institution for Illumina sequencing was UCR.

### Distribution of genes in the barley genome and its correspondence with rice syntenic regions

**Figure 1:**
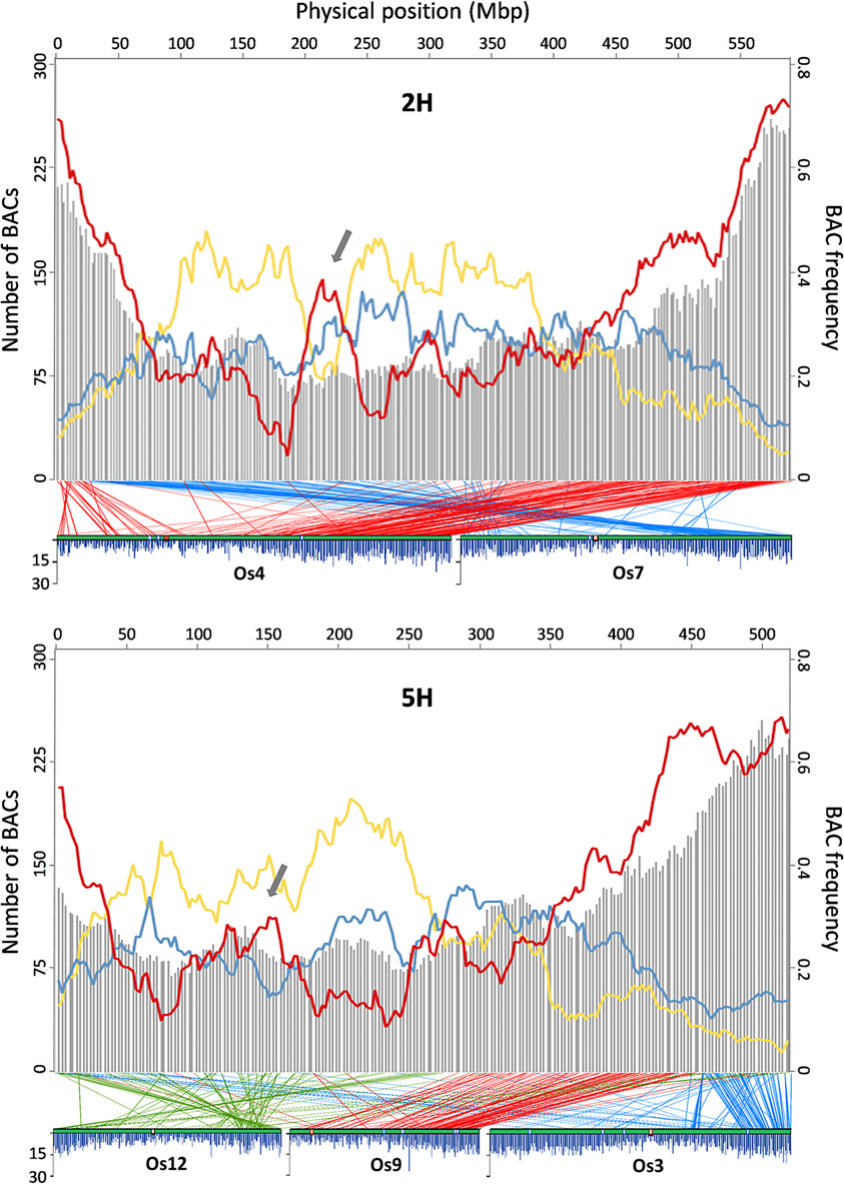
BAC distribution along barley chromosomes 2H and 5H and syntenic relationships with rice chromosomes. Grey bars represent the number of sequenced barley BACs and their units are shown on the left *Y*‐axis. Colored lines represent the proportion of BACs containing only one HC gene model (blue), three or more HC genes (red) or zero HC gene models (yellow), and the scale is shown on the right *Y*‐axis. BAC densities are calculated for a sliding window of 40 Mb at 2.5 Mb intervals based on the physical coordinates (archived golden path) provided by IBSC (2012). Gray arrows indicate gene‐dense regions different from distal ends. Barley–rice synteny is represented by lines connecting each mapped BAC to the position on the rice genome determined by BLASTX (see Experimental procedures). Densities of expressed rice genes across chromosomes are also displayed (adapted from Figure S2 in IRGSP, 2005), where blue bars indicate the frequency of gene models in 100 kb windows, red boxes indicate centromeres and white boxes represent physical gaps.

Most of the sequenced BACs (78%) were plotted across the barley genome based on the physical coordinates provided by IBSC (2012). The observed enrichment of clones towards the ends of the chromosomes was expected (Figures 1 and S2); it has been previously reported that distal regions of barley chromosomes have higher gene density than regions nearer to centromeres (IBSC, 2012). The sequences from these BACs allowed us to explore their gene content. On average we found 2.38 HC gene models per BAC, but some BACs did not contain any previously identified gene, while others contained over 10 HC genes. We further explored the distribution of BACs containing different numbers of genes along each chromosome. As shown in Figures 1 and S2, the location of BACs highly enriched with genes is clearly biased towards the distal ends of the chromosomes, but additional hotspots also exist (e.g. regions indicated by arrows in Figure 1). Conversely, the frequency of BACs containing zero or only one HC gene is lower toward the telomeric ends (Figures 1 and S2). Peaks of BACs containing zero genes are usually located in more central positions of the chromosomes. We note that BACs in the ‘gene‐bearing’ BAC list that have zero genes may have been false positives in the subjective scoring of library filter hybridizations. The uneven distribution of BACs containing at least three genes supports the idea of gene clustering that has been previously suggested for barley and other grass genomes (Barakat *et al*., 1997; Choulet *et al*., 2010; Gottlieb *et al*., 2013; Raats *et al*., 2013). In contrast with the variable BAC gene content, a uniform GC content was detected along all barley chromosomes, with chromosome averages ranging from 44.4% (3H and 7H) to 44.6% (4H) and an average GC content for all BACs of 44.5% (SD = 1.3%). This constant GC content was also found in wheat BACs located in different regions of 3B (Choulet *et al*., 2010) and in 1BS BAC‐end sequences (Raats *et al*., 2013). The BAC GC content percentages that we observe are similar to the GC composition of previously studied barley gene‐bearing BACs (Dubcovsky *et al*., 2001; Wei *et al*., 2002) and comparable to those of rye (Bartoš *et al*., 2008) and wheat genomes (Choulet *et al*., 2010; Raats *et al*., 2013). Although our dataset is biased toward gene‐containing BACs, we did not find any significant difference in GC content between gene‐rich and gene‐poor BACs.

To explore barley–rice synteny, each barley BAC DNA sequence was compared to rice translated gene models available at the Rice Genome Annotation Project database (http://rice.plantbiology.msu.edu/) using BLASTX (see Experimental procedures). The syntenic relationships between rice and barley that were revealed (Figures 1 and S2, lower plots) are consistent with previously reported observations (Stein *et al*., 2007; Close *et al*., 2009; Mayer *et al*., 2011). Single rice chromosomes (Os1 and Os2) are largely syntenic with barley 3H and 6H, respectively. Barley 5H has a more complex synteny with rice, sharing major syntenic regions with at least three rice chromosomes (Os3, Os9 and Os12). Each of the remaining barley chromosomes shares major blocks of conserved synteny with two rice chromosomes. In terms of gene content, we observed that the barley gene‐enriched chromosome regions tend to correspond to rice regions of high gene density (obtained from IRGSP, 2005). A clear example is barley chromosome 5H, where the gene‐dense portion extends further from the long‐arm distal end than in any other chromosome. This is the only distal region sharing synteny with gene‐enriched distal portions of two rice chromosomes. Indeed, the 5HL distal region shows two peaks of gene enrichment, which coincide with each of the two ancestors of rice chromosomes (Figure 1). In sharp contrast, the prominent peak that is visible in the central region of 2H does not have clear synteny to a gene‐rich region of the rice genome (Figure 1).

### Centromeric region of 4H

As mentioned above, 60 BACs were assigned to the region of overlap between the long and short flow‐sorted arms of chromosome 4H (4HC), some of which may be centromere clones. Only two of those BACs (0143O21 and 0474D04) contained a mapped Oligo Pool Assay (OPA) SNP (1_0424; Close *et al*., 2009), which was previously confirmed in the pericentromeric region of 4H (Muñoz‐Amatriaín *et al*., 2011). We also explored the gene content of these BACs. Only 14 BACs contain HC gene models when excluding highly frequent gene models (mostly TE‐related) (Table S3). This is an expected finding since centromeric regions are known to have low gene density. Although we did not find any annotated gene encoding the highly conserved centromere‐specific histone cenH3 (Zhong *et al*., 2002), we found genes previously identified in the centromere of rice chromosome 3 (ribosomal protein S5 and magnesium chelatase; Yan *et al*., 2006a) and a gene for retinoblastoma‐related protein, which has been recently shown to play an essential regulatory role in the assembly of cenH3 at Arabidopsis centromeres (Lermontova *et al*., 2013). Satellite sequences and centromere‐specific retrotransposons are the major constituents of centromeres in plants. While satellite repeats are problematic due to difficulties in the assembly of these tandem repeats, several retrotransposons including the conserved Ty3/gypsy type (Langdon *et al*., 2000; Hudakova *et al*., 2001) were found when inspecting highly frequent genes in these BACs (Table S3). These observations may provide useful leads for further studies of the centromere of barley chromosome 4H.

Although specific *k*‐mers were identified for centromeric regions of overlap of all other barley chromosomes except 1H (Ounit *et al*., 2015), none of our sequenced BACs was assigned to those regions. This is probably because, in 4H, the region shared between flow‐sorted short and long arms is larger than that in any other chromosome. However, when additional barley BACs are sequenced, it may be possible to identify centromeric BACs for other barley chromosomes. The use of analysis tools based on discriminative *k*‐mer (such as CLARK) in combination with chromosome arm sequence data could be used as an approach to define centromeric regions in other species where flow‐sorted arms exist (i.e. bread wheat).

### Identification of deviant genomic regions

**Figure 2:**
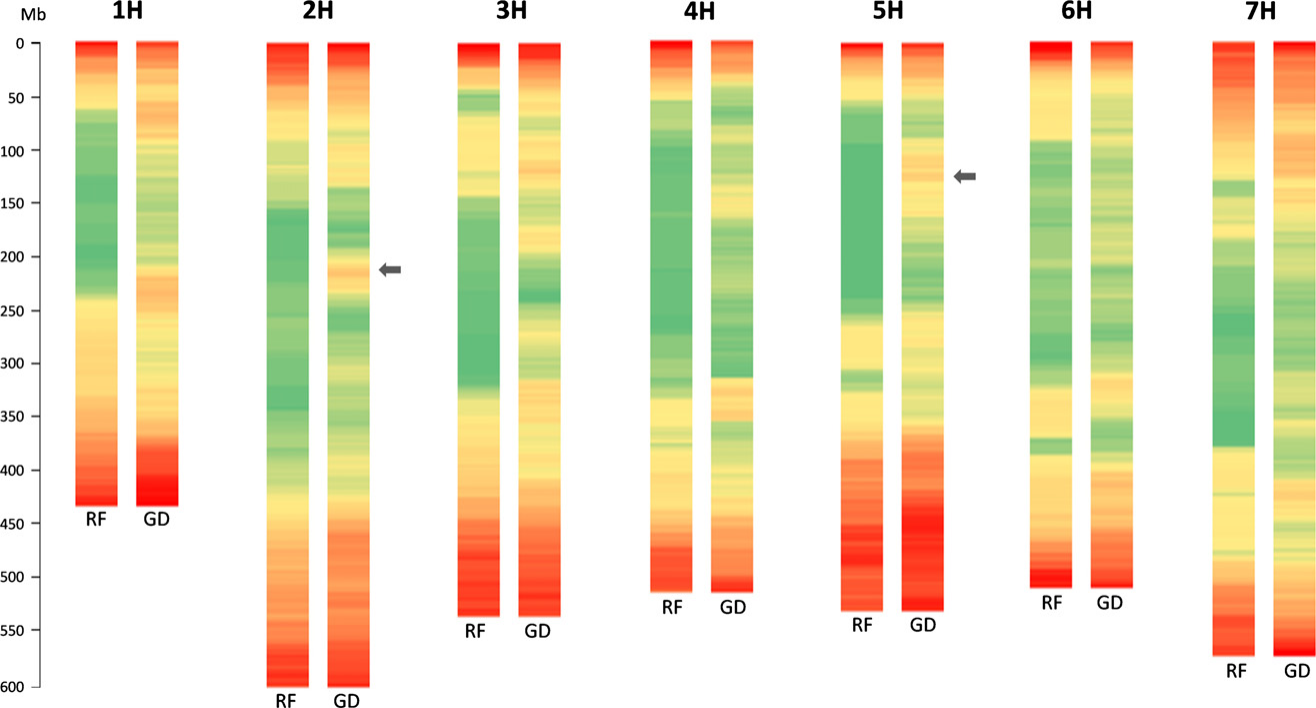
Relationship between recombination frequency (RF) and gene density (GD) along the seven barley chromosomes. Recombination rates are calculated from the cM/Mb ratios in sliding windows of 40 Mb with 2.5 Mb increments, and are represented by a color gradient from green (RF = 0) to red (RF = 1.14). Gene densities are estimated based on the total number of unique HC genes per window with respect to the total number of sequenced BACs assigned to that window, and are represented by the same color gradient from green (GD = 0.67) to red (GD = 3.16). Grey arrows indicate most evident genomic regions of relatively high gene density and very low recombination.

Triticeae chromosomes exhibit an increase in gene density and recombination rate along the centromere‐telomere axis (Dvorák, 2009). This general trend has been observed in sequence data from barley (IBSC, 2012; Zeng *et al*., 2015), wheat (Raats *et al*., 2013; Choulet *et al*., 2014), and *Ae. tauschii* (Luo *et al*., 2013), and it is common to other grasses (i.e. *Brachypodium*, rice, and maize). The possibility of examining larger pieces of genome sequence (i.e., BACs) accounting for one third of the barley genome allowed us to take a closer look at how recombination rate distribution relates to gene distribution. As shown in Figure 2, recombination frequency generally increases along the centromere–telomere axis. Similarly, gene density distribution is not uniform along the chromosomes and it is usually correlated with recombination frequency (i.e., higher in distal ends). However, we also observe regions that clearly deviate from these general characteristics; there are regions with relatively high gene density embedded within areas with suppressed recombination. The two clearest examples are in chromosomes 2H and 5H (Figure 2, grey arrows). The 2H region coincides with the prominent peak of gene‐rich BACs shown in Figure 1 and comprises ~18 Mb. A total of 50 BACs containing 84 HC unique gene models (Table S4) are located in this region of 2H. GO‐term enrichment analyses performed in agriGO (Du *et al*., 2010) using GO terms available from MIPS (http://pgsb.helmholtz-muenchen.de/plant/barley/; Nussbaumer *et al*., 2013) revealed a slight enrichment for genes belonging to category ‘cellular component organization,’ including alpha‐tubulins, peptide chain release factors, and a thylakoid‐formation protein (thf1) (Table S4), which perform essential cellular functions. This barley region shares conserved synteny with the *Ae. tauschii* 2D region extending from 168 to 182 Mb in the Luo *et al*. (2013) physical map (ctg3995, ctg157 and ctg3482).

The 5H region is much larger, extending for approximately 60 Mb, including 136 BACs and 174 HC gene models. Interestingly, the GO category ‘response to biotic stimulus’ was a highly overrepresented. A set of seven genes belonging to the ‘Bet v I type allergen’ family of proteins is responsible for this enrichment. Different pollen allergens and pathogenesis‐related proteins (i.e. STH‐21; Table S4) are included in this family. Due to their homology with pathogenesis‐related proteins, Bet v I pollen allergens are considered to be involved in pathogen resistance of pollen (Breiteneder *et al*., 1989). This recombination cold‐spot did not contain any of the rapidly evolving nucleotide binding, leucine‐rich‐repeat (NLR) encoding R genes, which tend to cluster in high‐recombination distal regions of the barley chromosomes (IBSC, 2012; Muñoz‐Amatriaín *et al*., 2013). The other enriched GO category in the highlighted 5H region was ‘DNA replication,’ involving genes with more conserved functions. Some barley BACs in this region are syntenic to *Ae. tauschii* sequences at 94 Mb on 5D (ctg582; Luo *et al*., 2013).

A list of HC genes located in the aforementioned regions is provided in Table S4. To facilitate the exploration of these and other regions of the barley genome, Data S2 contains the recombination frequency and gene density data corresponding to Figure 2 with detailed information of barley physical and genetic positions. As recombination determines the extent of linkage disequilibrium (LD), it impacts both genome‐wide association studies (GWAS) and genomic selection (GS). Regions of high LD result in spurious marker‐trait associations in GWAS. Modification of GS models to emphasize recombinants in low‐recombination regions can be particularly important in regions with higher gene content where genetic load may also be higher (Morrell *et al*., 2012). Similarly, marker‐assisted trait introgression and map‐based cloning are affected by recombination, since large populations may be required to break linkage drag or to find markers within a short physical distance of the target gene. Thus, knowledge of recombination rates and gene densities provided in this work is of relevance for the effective use of genetic variation in barley and related species.

### HarvEST:Barley allows easy access to GB‐BAC sequence assemblies and synteny with *Ae. tauschii*

To successfully deploy genome sequence information for crop improvement, it is critical to make it easily accessible. We have developed an online interface to provide access to the latest sequence assemblies (v.4.1) from 15 622 MTP gene‐bearing BACs (http://harvest-web.org/hweb/utilmenu.wc). BAC sequences and their annotations can be retrieved by ‘BAC address,’ which can be obtained by BLAST via http://www.harvest-blast.org/ using the user's input sequence. Since high‐throughput SNP genotyping (Close *et al*., 2009; Comadran *et al*., 2012) is routinely used in modern barley breeding, genome data can also be downloaded using a ‘SNP name’ as a query. Alternatively, HarvEST:Barley (http://harvest.ucr.edu/) may be installed on a personal computer to export BAC sequences, gene annotations and both physical and genetic map coordinates in a similar manner, with additional options of exporting BACs by chromosome arm or genetic map interval. HarvEST:Barley is available for Microsoft Windows as a single 1.3 GB installation file and does not require an internet connection to operate. These two interfaces also provide the option of accessing information from only the specified BACs or all BACs in the same contig.

Along with the 15 622 BAC sequences described in the present work, the databases noted above contain sequences from additional BACs from the Yu *et al*. (2000) library. This includes: 3153 BAC assemblies generated using 454 sequencing (IBSC, 2012); 50 BACs fully sequenced at the Joint Genome Institute by Sanger sequencing; and 21 BAC sequences available from the National Center for Bioinformatics (NCBI) database. A list of these BACs sequenced by other methods, some of which coincide with the BACs that we sequenced, can be found in Data S3.

The latest version of HarvEST:Barley has implemented a barley–*Aegilops tauschii* synteny viewer, enabling facile comparisons between barley and this diploid ancestor of the wheat D‐genome. The map‐anchored SNP design sequences from *Aegilops tauschii* (Luo *et al*., 2013) were compared with our BAC sequences to relate barley BACs to orthologous positions in wheat linkage groups. Similarly, barley SNP design sequences were used to anchor barley BACs to barley linkage groups. The net result was to cross‐relate barley and *A. tauschii* via the sequenced BACs (see Experimental procedures for more information). We found clear synteny between barley and *Ae. tauschii* (Figure S3) in every linkage group. In addition, several substantial inversions were identified on chromosomes 1H (69.7–74.1 cM and 104.1–114.9 cM), 2H (21.7–26.9 cM and 84.5–88.0 cM), 3H (11.0–20.03 cM and 86.9–103.8 cM), and 4H (98.7–102.4 cM). This synteny browser is in addition to the previously available barley–rice and barley–*Brachypodium distachyon* synteny displays within HarvEST:Barley, all of which can be retrieved by selecting either of the two latest barley genetic consensus maps (Muñoz‐Amatriaín *et al*., 2011, 2014).

## Conclusions

Accessing the entire genome sequence of the economically important crop and the Triticeae genetic model, barley, has proven difficult due to its large and highly repetitive genome. Here we identified regions that contain genes by screening a BAC library made from the reference genotype ‘Morex’ using genic probes, followed by BAC‐clone sequencing. Analysis of the ~1.7 Gb of gene‐rich genomic sequence corresponding to 15 622 ‘gene‐bearing’ BACs shows that in addition to distal ends of chromosomes containing most of the gene‐enriched regions, gene‐rich islands exist in more interior positions of the chromosomes. Interestingly, some of these interior islands are in areas with suppressed recombination, which has both practical and evolutionary implications. Another outcome of this work was to identify several BACs from chromosome 4H that may include the actual centromere. The method of defining arm‐specific *k*‐mers that led to this outcome is broadly applicable to other species where flow‐sorted chromosome arms are available. Access to these BAC sequences and their annotations is facilitated through an online interface and the software HarvEST:Barley, which additionally contains a barley–*Aegilops tauschii* synteny viewer. We found a highly syntenic relationship between these two species, except for a few clear inversions on chromosomes 1H‐4H. This study constitutes an additional resource for the Triticeae community that will improve the speed and precision of map‐based cloning and marker development while supporting ongoing efforts to obtain a complete reference sequence of barley.

## Experimental Procedures

### Identification of gene‐containing BACs

A 6.3 ×  haploid‐genome‐equivalent barley BAC library (Yu *et al*., 2000) was obtained as library filter sets from Clemson University Genomics Institute, as were rearrayed cultures of BAC clones used for fingerprinting. This library was constructed using *Hind*III partially digested cultivar Morex DNA ligated to pBeloBAC11 vector and arrayed on 17 filters, each with 18 432 clones. Compilation of 81 831 gene‐bearing BACs was accomplished in two ways. In total, 21 689 BACs were identified from information provided by several researchers based on their prior work (Data S1). In addition, oligonucleotide probes (‘overgos’) designed mainly from transcript sequences (EST unigenes) were used in hybridizations to identify 72 141 putative gene‐bearing BACs. The union of these two sets was 83,831 unique GB‐BACs. Information regarding the pools of probes used for BAC identification and details of the hybridization process can be found in Method S1.

As the hybridizations using pools of overgos progressed, we observed grsadually fewer newly identified BACs per hybridization. The frequency of new BACs identified for each of the large pools of probes generally diminished from a starting range of 60–85% to a range of 27–57%. To provide an estimate of the percentage of all possible gene‐bearing BACs identified in this work, the hybridization data were randomly shuffled and sampled 10 000 times to plot the number of unique BACs identified as a function of the number of probe pools applied. Extrapolation provided an estimate of the number of pools that would be necessary to approach the asymptotic limit of the number of gene‐bearing BACs. From this treatment of the data, we estimated 107 882 gene‐bearing BACs in the 6.3 ×  Morex library (Figure S4), which is roughly 1/3 of all BACs genome‐wide. However, the nature of our BAC detection method (genic probes) made it more likely that we would find a BAC containing multiple genes than a BAC carrying only one gene, and as a consequence the asymptotic limit could be higher than indicated by this extrapolation.

### BAC‐clone fingerprinting and assembly

BAC DNA was isolated and fingerprinted according to Luo *et al*. (2003). BAC clones maintained in a 384‐well plate were inoculated into four 96‐deep well plates containing 1.2 mL 2 ×  YT medium with 12.5 μg ml^−1^ chloramphenicol and grown at 37°C for 24 h. BAC DNA was isolated with the Qiagen R.E.A.L 96‐Prep kit (https://www.qiagen.com/us/shop/sample-technologies/dna-sample-technologies/plasmid-dna/real-prep-96-plasmid-kit/) either manually or by Qiagen robot. 0.5–1.2 μg of DNA was then digested with 5 units each of *Bam*HI, *Eco*RI, *Xba*I, *Xho*I and *Hae*III enzymes and transferred to SNaPshot multiplex labeling solution. Capillary electrophoresis was performed on the DNA using ABI internal size standard LIZ‐500 (35–500 bp) in an ABI 3730 DNA sequencer (Applied Biosystems, USA; http://www.thermofisher.com/us/en/home/brands/applied-biosystems.html). GenoProfiler software (You *et al*., 2007) was used for automated editing of sized fingerprinting profiles generated by the ABI Genetic Analyzers (Applied Biosystems). The batch‐processing module extracts sized fragment information either directly from the ABI raw trace files or from data files exported from GeneMapper (Applied Biosystems) or other size calling software, removes background noise and undesired fragments, and generates fragment size files. High‐throughput fingerprinting was attempted for 83 831 clones. In total, 2967 clones (5%) were deleted during fingerprint editing due to lack of insert, failed fingerprinting, or being ignored by the GenoProfiler software. Clones containing four or fewer fragments in the range of 50 to 500 bp provided insufficient information to be included in contig assembly. The clones from this library had an average of 92 fragments per clone in the sized range.

BAC clones were assembled into contigs using a compartmentalized approach (Bozdag *et al*., 2009). Each group of BACs identified by each probe pool was first assembled independently using Fingerprint Contigs (FPC) software v8.1 (Soderlund *et al*., 1997, 2000). Contig merging and redundancy removal steps were then applied (Bozdag *et al*., 2009) to finalize the assembly. Key elements of this compartmentalized process can be found in Methods S1.

### Construction of minimal tiling paths

Two sets of MTP clones were computed. The first one contained 2638 BACs from data that were available relatively early in this work, including the majority of GB‐BACs provided from prior work and GB‐BACs containing abiotic‐stress regulated genes and others targeted by a SNP genotyping assay referred to as POPA1 (Close *et al*., 2009). These BACs are referred to later as MTP subsets 1‐3. Subsequently, after the full list of 83 831 GB‐BACs had been compiled, the second set of MTP clones, 13 182 BACs, was developed. BACs from this second MTP set, which generally avoided duplication with the first set of MTP clones, are referred to later as MTP subsets 4–9.

### MTP sequencing and assembly

MTP BACs were paired‐end sequenced (2 × 100 bases) using Illumina HiSeq2000 (Illumina, Inc, San Diego, CA, USA; https://www.illumina.com/). Sequencing was done in eight sets of BAC clones (HV3‐10) applying a combinatorial pooling design (Lonardi *et al*., 2013). In brief, this approach takes advantage of the current high‐throughput sequencing instruments to *de novo* sequence thousands of BAC clones in pools that are designed to identify each BAC within the pooling pattern, hence avoiding exhaustive DNA barcoding. Reads in each pool (after quality trimming) were ‘sliced’ into smaller samples of optimal size as explained in detail by Lonardi *et al*. (2015), deconvoluted, and then assembled BAC‐by‐BAC using Velvet v.1.2.09 (Zerbino and Birney, 2008). Assembly of the BACs with SPAdes (Bankevich *et al*., 2012) or IDBA (Peng *et al*., 2012) did not provide a clear objective improvement in assembly quality. Slicing the data into subsets was a key step to improving the quality of the assemblies. A threshold cutoff of 35 000 high‐quality reads was applied to consider a BAC as ‘sequenced’. Statistics for assembly were collected using the Assemblathon script (K. Bradnam, Genome Center, UC Davis, USA).

High confidence (HC) and low‐confidence (LC) gene models predicted by IBSC (2012) were used to annotate the BAC assemblies, using a minimum sequence length of 200 bp and an e‐value of 1e^−20^ as the cutoffs for the BLAST alignments. We ignored any gene model hitting at least 10 BACs for most analyses.

### Chromosome arm assignment of BACs

We used CLARK, a supervised classification method, to assign BACs to chromosome arms (Ounit *et al*., 2015) Briefly, CLARK can accurately classify ‘objects’ (e.g., BACs) to ‘targets’ (e.g., chromosome arms) by reducing the problem to a *k*‐mer comparison of the corresponding sequences. CLARK differs from other *k*‐mer based methods because it considers only *k*‐mers that are specific (or discriminative) to each target. It does so in the preprocessing phase by discarding any *k*‐mer that appears in two or more targets, except in the case of *k*‐mers shared by only both arms of the same chromosome, which are used to define ‘centromeric’ regions of overlap. Additionally, CLARK disregards very rare *k*‐mers, which tend to be spurious from sequencing errors. Using *k *= 19 and by discarding 19‐mers that appeared only once (Ounit *et al*., 2015), we have accepted only assignments with confidence scores >0.75 (HC assignments). ‘Targets’ were reads generated by Illumina WGS sequencing of barley flow‐sorted chromosome 1H and arms of chromosomes 2H–7H that were assembled using SOAPdenovo (Luo *et al*., 2012). The chromosomes were purified by flow cytometric sorting as described by Suchánková *et al*. (2006) and their DNA amplified following the procedure of Šimková *et al*. (2008).

### Validation of the sequence assembly

A total of 1037 gene‐bearing BACs from the Yu *et al*. library (2000) were previously sequenced (454 Life Sciences technology; http://www.454.com/) and assembled by other institutions, and included in the barley genome sequence resource (IBSC, 2012). Our sequence assemblies for 1037 BACs of the same address BACs were compared using the sequence alignment tool QUAST (Gurevich *et al*., 2013). We removed 40 BACs which had <33% alignment with each other, attributing this level of disagreement to rearray errors, extensive cross‐contamination or extreme instability of a BAC in the *Escherichia coli* host. The remaining 997 BACs that were sequenced independently in the present work and previously were blasted against HC and LC gene models predicted by IBSC (2012), using a minimum sequence length of 200 bp and an e‐value of 1e^−20^. Gene models found in our sequence assemblies and in the 454‐based assemblies were compared, after excluding gene models hitting at least 10 BACs (Table 2). Fourteen of the 50 BACs that were fully sequenced by the Joint Genome Institute co‐authors using the Sanger method were in common with the 15 622 BACs and were used as ground truth for additional validation of the sequence assemblies. QUAST (Gurevich *et al*., 2013) was used to determine the percentage coverage. Gene content was determined as described above.

### Synteny analysis

For barley–rice synteny, each barley BAC DNA sequence was compared to rice translated gene models available at the Rice Genome Annotation Project database (http://rice.plantbiology.msu.edu/). All BLASTX hits with an e‐value of −20 or better were tallied for each BAC. The rice chromosome with the plurality of matches was then taken as the correct rice chromosome. A mean value of chromosome coordinates was then calculated for the matched rice gene models on this chromosome to assign a rice chromosome position to each BAC. A rice genome position was then assigned to each entire BAC contig by a similar voting method, accepting the plurality of rice chromosomes for the contig and the mean value within the matching rice chromosome as the position of the BACs in this contig. These rice genome coordinates were then used to align the seven barley chromosomes with the 12 rice chromosomes (Figures 1 and S2).

For barley–*Aegilops tauschii* synteny, each SNP design sequence for the *Ae. tauschii* iSelect genotyping assay (Luo *et al*., 2013), downloaded from http://probes.pw.usda.gov/WheatDMarker/downloads/, was matched by BLAST to the extended *Ae. tauschii* genome sequences that were available from the same website. The linkage group and cM coordinates for each SNP marker published in Luo *et al*. (2013) were then associated with each wheat D‐genome iSelect SNP assay design sequence. These wheat SNP design sequences were matched by BLAST to the sequences of barley BACs described in the present work, many of which contained sequences matching barley SNP assay design sequences (Close *et al*., 2009; Comadran *et al*., 2012). The arm assignment for each barley BAC was taken from Data S1, to limit the BACs to those where the barley and wheat SNPs mapped to orthologous chromosomes. The barley–wheat D synteny viewer in HarvEST:Barley is based on these relationships.

## Accession numbers

The BAC sequence assemblies supporting the results of this article are available in NCBI under accession numbers: AC250421 to AC252610, AC256303 to AC269749, AC256237 to AC256288, AC250484, AC250784, AC250371, AC251557, AC251639, AC251663, AC251805, AC251814, AC252228, AC252453, and AC252497. Flow‐sorted chromosome arm sequences can be found at NCBI under accession no. SRX143974.

## Supporting information


**Figure S1.** Scatter plot of number of gene‐bearing sequenced BACs against molecular sizes for barley chromosome arms.

**Figure S2.** BAC distribution along barley chromosomes 1H, 3H, 4H, 6H and 7H.

**Figure S3.** Synteny between barley and *Ae. tauschii* linkage groups.

**Figure S4.** Estimate of the total number of gene‐bearing BACs.

**Table S1.** Statistics of BAC sequence assembly for different minimum node sizes.

**Table S2.** High confidence (HC) and low‐confidence (LC) gene models predicted by IBSC (2012) that hit ≥10 BACs.

**Table S3.** BAC clones assigned to 4HC.

**Table S4.** HC gene models located in gene‐dense and low‐recombination regions of 2H and 5H.

**Methods S1.** Supplementary methods and full legends for supporting information.

**Data S1.** List of gene‐bearing BACs identified from the HVVMRXALLhA library (Yu *et al*., 2000), and the genic probe pool(s) that detected each of those 83,831 BACs.

**Data S2.** Recombination frequency and gene density data corresponding to Figure 2.

**Data S3.** List of BAC clones from the Yu *et al*. (2000) library sequenced by institutions other than UCR and whose sequences have been added to HarvEST:Barley.
