# Supplementary figures and images for: Sequencing of 15 622 gene‐bearing BACs clarifies the gene‐dense regions of the barley genome
Source: Plant J. 2015 Sep 21;84(1):216–27. doi: 10.1111/tpj.12959 (PMC5014227; doi:10.1111/tpj.12959)

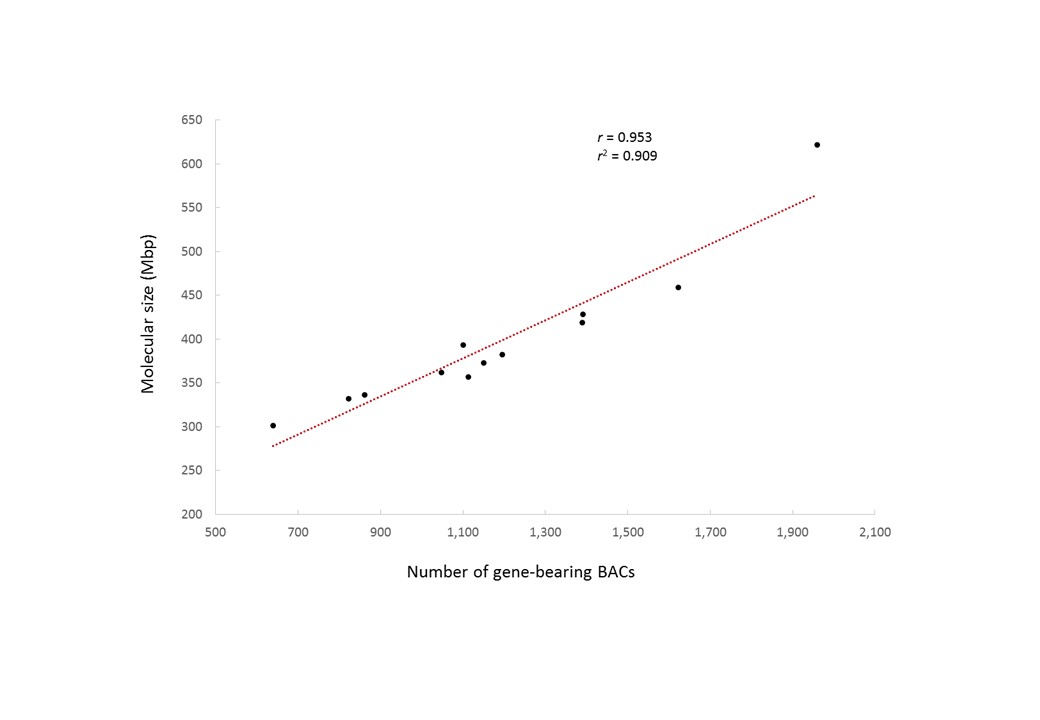

Supplement: Supplementary file 1 — Figure S1. Scatter plot of number of gene‐bearing sequenced BACs against molecular sizes for barley chromosome arms. [file TPJ-84-216-s001.jpg]

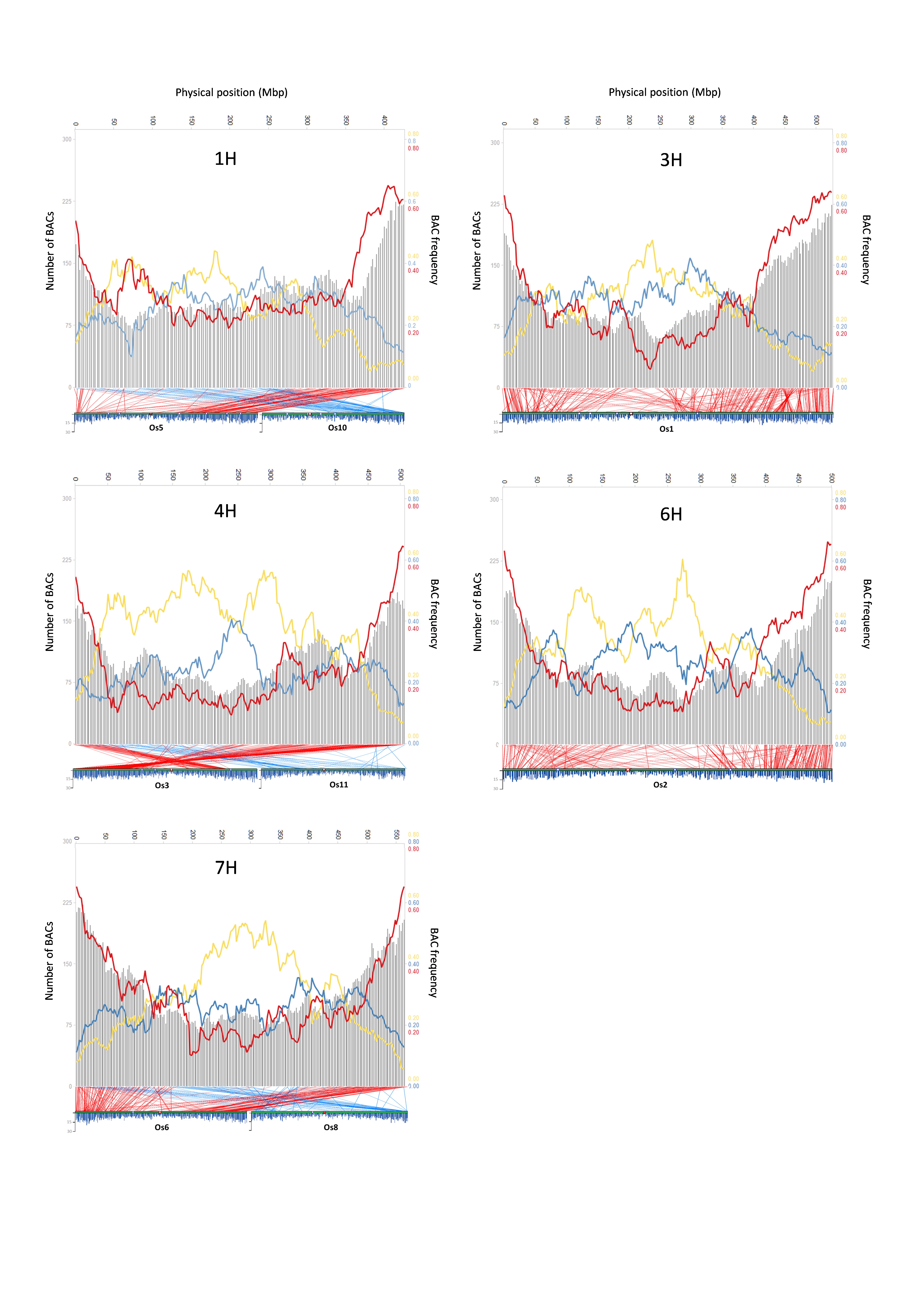

Supplement: Supplementary file 2 — Figure S2. BAC distribution along barley chromosomes 1H, 3H, 4H, 6H and 7H. [file TPJ-84-216-s002.jpg]

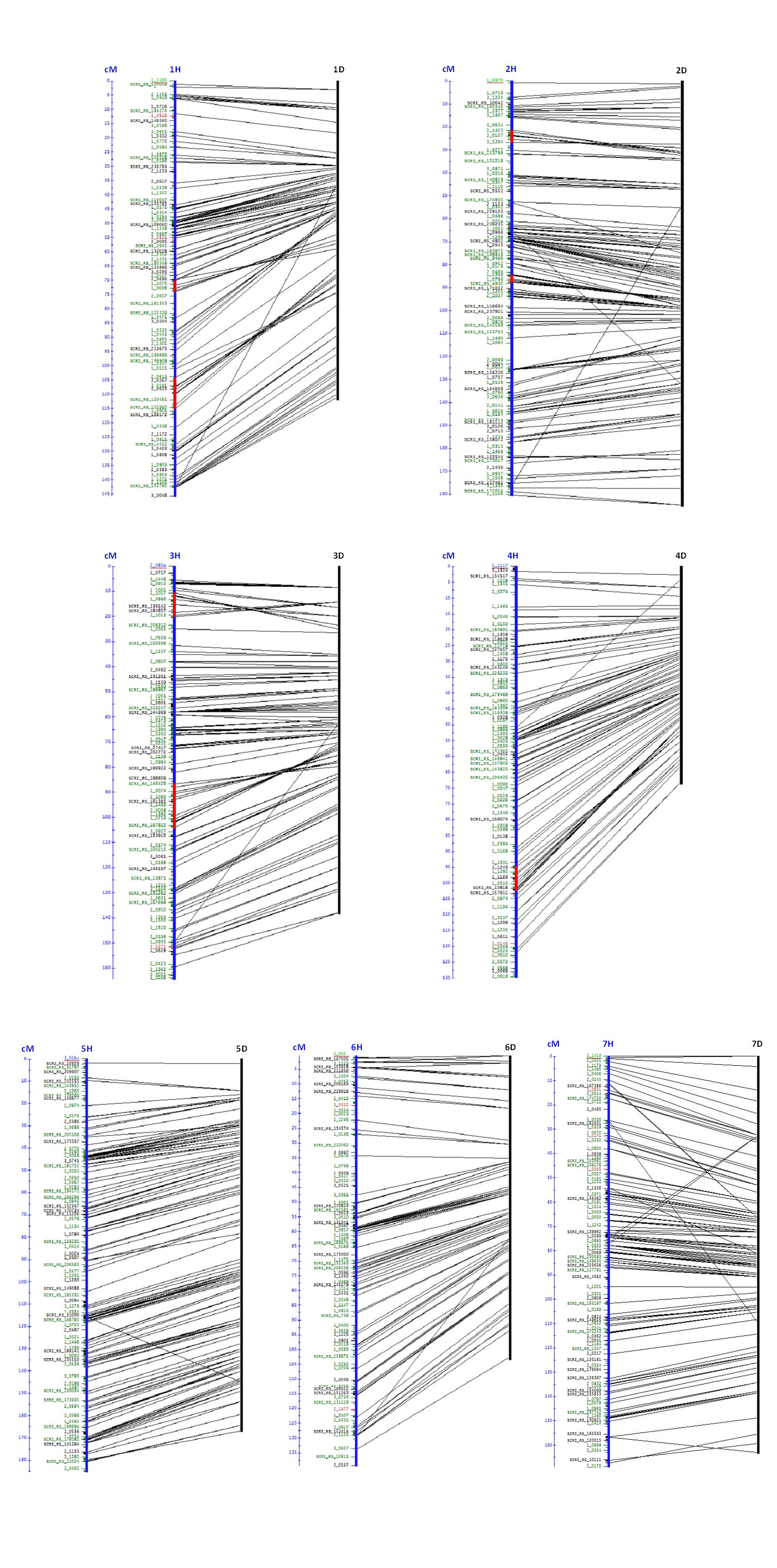

Supplement: Supplementary file 3 — Figure S3. Synteny between barley and Ae. tauschii linkage groups. [file TPJ-84-216-s003.jpg]

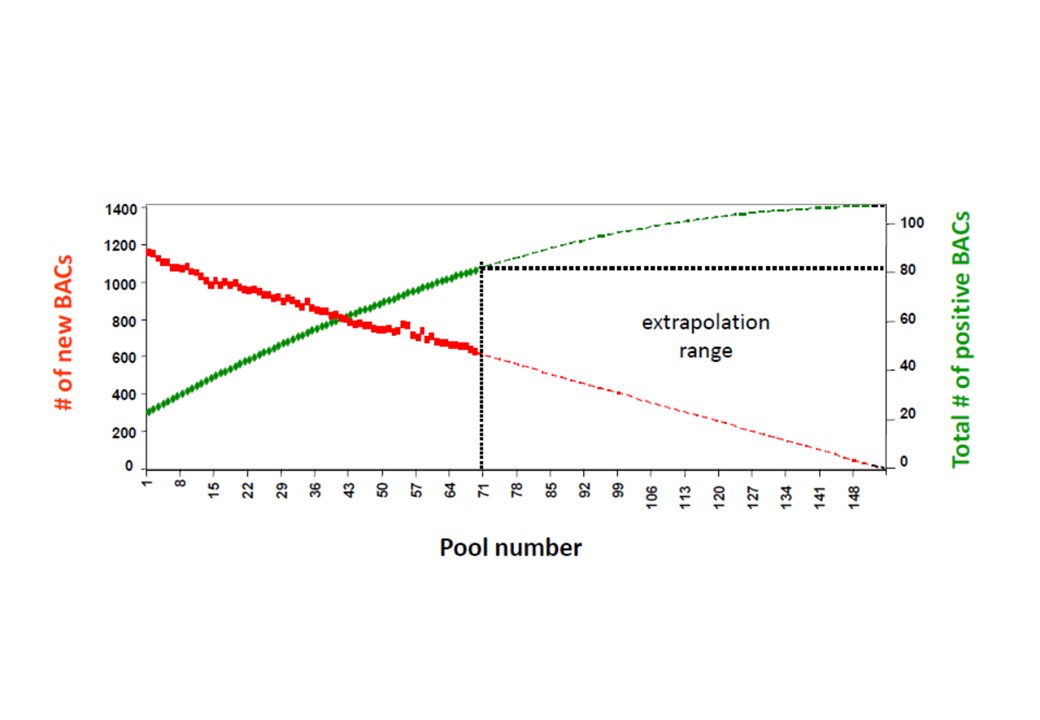

Supplement: Supplementary file 4 — Figure S4. Estimate of the total number of gene‐bearing BACs. [file TPJ-84-216-s004.jpg]
