# Supplementary material for: Sequencing of 15 622 gene‐bearing BACs clarifies the gene‐dense regions of the barley genome
Source: Plant J. 2015 Sep 21;84(1):216–27. doi: 10.1111/tpj.12959 (PMC5014227; doi:10.1111/tpj.12959)
Supplement: Supplementary file 9 — Methods S1. Supplementary methods and full legends for supporting information. [file TPJ-84-216-s009.docx]

**Supplementary methods**

**Information on probes used for GB-BAC identification.** The algorithms that were used to design genic overgo probes were provided in Zheng et al. (2006) and are available through the OligoSpawn interface at http//www.oligospawn.org. Details of the overgo labeling and hybridization procedures were provided by Madishetty et al. (2007). Hybridization pools c1 through c69 included a total of 12,285 probes, of which 12,059 were intended to find a single gene and 226 were intended to find several to many genes sharing the probe sequence (“popular” probes). In general, these probes were designed to find genes in functional categories, or by expression pattern, or location on a specific chromosome. Most of the probes that were chosen by their expression pattern made use of experiments conducted using the Barley1 GeneChip (Close et al. 2004), especially drought stress, low temperature, salinity or abscisic acid application. Probes addressing functional categories included transcription factors, photosynthetic processes, kinases, phosphatases, cell wall biogenesis and numerous others. The overgos in pools c4 through c9 were selected from a list of “popular” oligonucleotides (Zheng et al. 2006) to maximize the number of gene-positive BACs per probe, but in some cases problems were encountered with highly repetitive sequences. The first few pools (c1 through c3) were composed of 40 bp overgos corresponding to genes indicated by the literature as pertinent to abiotic stress; the remainder of the overgos produced 36 bp probes. The nature of pools c0 and all other probes from researchers who provided GB-BAC addresses from prior work varied widely, including cDNAs, genomic DNA fragments, overgos and PCR amplification.

**Hybridization process.** Autoradiographs were analyzed using High Density Filter Reading (HDFR) software from Incogen Inc. (Williamsburg, VA). X-ray films were scanned and imported into HDFR, where a grid file was generated for the filter layout reflecting the 18,432 clone addresses. Filter images were aligned with the grid using the background and a few (3-4) strong signal positive BACs, then each filter was scored and positives compiled into a text file for each pool. Each BAC was spotted at two locations within a 4 x 4 grid in a unique pattern to facilitate correct scoring of positive clones. All filter images were scored by a second, and sometimes a third, person. Any BAC scored positive by any person was added to the list of GB-BACs, with the expectation that this would result in some false positives since each person applied subjective judgment as to the boundary of positive versus negative hybridizations.

**Compartmentalized assembly method.** Contigs were assembled using a tolerance of 3 and a cutoff of 1e^-45^ with all other parameters at default values. FPC’s End-Merger function was applied in several iterations with a cutoff of 1e^-40^. To avoid making wrong merges early in the process, End-Merger was run with increasingly lower values of the “match” parameter (the required number of matching clones in one of the ends of the contigs that will be merged) (6 for the first iteration, 4 for the second iteration, and 3 for subsequent iterations). Cutoff values of 1e^-50^, 1e^-55^, 1e^-60^ were used iteratively to resolve Q-clones. Q-contigs (contigs that contain at least one Q-clone) that contain 15% or more Q-clones were then split into component parts to decrease the number of Q-clones. To merge contigs that share many clones, a similarity probability was computed (the probability that two contigs share a set of clones by chance) as per Bozdag et al. (2009) and then contigs that have a similarity probability less than a threshold were merged using Merge-Similar-Contigs software. A threshold of 0 was used for the first iteration, 1e^-30^ for the second iteration, and 1e^-15^ for subsequent iterations. After the automatic assembly of the fingerprinted clones, there were 72,052 clones, 10,794 contigs, 10,598 singletons, and 996 Q-contigs. Only 75 of these Q-contigs contain 15% or more Q-clones.

**References**

Bozdag, S., Close, T.J., and Lonardi, S. (2009) A compartmentalized approach to the assembly of physical maps. *BMC Bioinformatics* **10**, 217.

Close, T.J., Wanamaker, S., Caldo, R.A., Turner, S.M., Ashlock, D.A., Dickerson, J.A., Wing, R.A., et al. (2004) A new resource for cereal genomics: 22K barley GeneChip comes of age. *Plant Physiology* **134**, 960-968.

Madishetty, K., Condamine, P., Svensson, J.T., Rodriguez, E., and Close, T.J. (2007) An improved method to identify BAC clones using pooled overgos. *Nucleic Acids Res*. **35**, e5-e7.

Zheng, J., Svensson, J.T., Madishetty, K., Close, T.J., Jiang, T., and Lonardi, S. (2006) OligoSpawn: a software tool for the design of overgo probes from large unigene datasets. *BMC Bioinformatics* **7**, 7.
