# Supplementary material for: Sequencing of 15 622 gene‐bearing BACs clarifies the gene‐dense regions of the barley genome
Source: Plant J. 2015 Sep 21;84(1):216–27. doi: 10.1111/tpj.12959 (PMC5014227; doi:10.1111/tpj.12959)
Supplement: Supplementary file 13 [file TPJ-84-216-s013.docx]

**Legends for supporting information**

**Figure S1.** Scatter plot of number of gene-bearing sequenced BACs against molecular sizes for barley chromosome arms. The regression line, correlation coefficient (*r*) and coefficient of determination (*r*2) are shown. Molecular sizes of barley chromosome arms were extracted from Suchánková et al. (2006).

**Figure S2.** BAC distribution along barley chromosomes 1H, 3H, 4H, 6H and 7H. Grey bars represent the number of sequenced barley BACs and their units are shown on the left Y-axis. Colored lines represent the proportion of BACs containing only 1 HC gene model (blue), 3 or more HC genes (red) or 0 HC gene models (yellow), and the scale is shown on the right Y-axis. BAC densities are calculated for a sliding window of 40 Mb at 2.5 Mb intervals based on the physical coordinates provided by IBSC (2012). Barley-rice synteny is represented by lines connecting each mapped BAC to the position on the rice genome determined by BLASTX (see Experimental procedures). Densities of expressed rice genes across chromosomes are also displayed (adapted from Supplementary Figure 2 in IRGSP (2005) where blue bars indicate the frequency of gene models in 100 kb windows, red boxes indicate centromeres and white boxes represent physical gaps).

**Figure S3.** Synteny between barley and *Ae. tauschii* linkage groups. The connecting lines indicate a relationship between *Ae. tauschii* SNP design sequence and a barley SNP design sequence, the relationship being the presence of both within an assembled barley BAC sequence (see Experimental procedures and text for further details). Genetic map positions are based on the consensus map available from Muñoz-Amatriaín et al. (2014).

**Figure S4.** Estimate of the total number of gene-bearing BACs. The X-axis represent the probe pool number starting with pool 1. The hybridization data were randomly shuffled and sampled 10,000 times to plot the number of unique BACs identified as a function of the number of probe pools applied. The left vertical axis represents the mean number of newly identified BACs (in red, declining linearly), while the right vertical axis show the mean value of the total number of unique BACs identified (in green, increasing asymptotically). Linear extrapolation of the mean values of new BACs is shown as dashed lines.

**Table S1.** Statistics of BAC sequence assembly for different minimum node sizes. (*) Numbers do not include gene models hitting >= 10 BACs. (NA) numbers of gene models not shown for these node sizes as a niminum length of 200 bp was used for the BLAST alignments.

**Table S2.** High-confidence (HC) and low-confidence (LC) gene models predicted by IBSC (2012) that hit ≥ 10 BACs.

**Table S3.** BAC clones assigned to 4HC. HC gene models contained in those BACs are shown. Gene models hitting ≥10 BACs are indicated with an asterisk.

**Table S4.** HC gene models located in gene-dense and low-recombination regions of 2H and 5H.

**Methods S1. Supplementary methods and full legends for supporting information.**

**Data S1**. List of gene-bearing BACs identified from the HVVMRXALLhA library (Yu et al. 2000), and the genic probe pool(s) that detected each of those 83,831 BACs. The letter preceding the probe pool number corresponds to the research group that designed the probe (a=Young Gu; b=Kulvinder Gill; c=Timothy Close; d=Jorge Dubkovsky; e=David Laurie; f=Anders Falk; g= Perry Gustafson; h=Patrick Hayes; i=Nick Collins; k=Andy Kleinhofs; l=Tom Blake; m=Gary Muehlbauer; n=Saghai Maroof; p=Wayne Powell; s=Nils Stein; t=Katherine Feuillet; u=Takao Komatsuda; w=Roger Wise; z=Unknown probe). Contig numbers for BACs that were effectively fingerprinted and assembled are indicated on the table. Additionally, sequencing information of gene-bearing BACs identified as minimal tiling path (GB-MTP) is shown. Column I indicates if an institution other than UCR sequenced the BAC.

**Data S2.** Recombination frequency and gene density data corresponding to Figure 2. For each window, information of physical positions (Archived Golden Path coordinates in IBSC (2012)) and cM positions from different genetic maps is also added to facilitate the use of the data. (*) cM position base on the latest consensus genetic map published in Muñoz-Amatriaín et al. (2014).

**Data S3.** List of BAC clones from the Yu et al. (2000) library that have been sequenced by institutions other than UCR and whose sequences have been added to HarvEST:Barley. Some of these BACs are part of our MTP gene-bearing BAC set and, hence, have been also sequenced as part of this study. IBSC=sequences published in IBSC (2000); JGI=sequenced at the Hudson Alpha Institute using Sanger sequencing; NCBI=complete BAC sequences downloaded from GenBank).
